# Optimal Model Mapping for Intravoxel Incoherent Motion MRI

**DOI:** 10.3389/fnhum.2021.617152

**Published:** 2021-02-22

**Authors:** Yen-Peng Liao, Shin-ichi Urayama, Tadashi Isa, Hidenao Fukuyama

**Affiliations:** ^1^Division of Neurobiology and Physiology, Department of Neuroscience, Graduate School of Medicine in Kyoto University, Kyoto, Japan; ^2^Human Brain Research Center, Graduate School of Medicine in Kyoto University, Kyoto, Japan; ^3^Faculty of Medicine, Institute for the Advanced Study of Human Biology (ASHBi), Kyoto University, Kyoto, Japan; ^4^Department of Rehabilitation Medicine, Graduate School of Medicine, Nagoya City University, Nagoya, Japan

**Keywords:** IVIM-MRI, perfusion, diffusion, modeling, gaussian, kurtosis, gamma, AIC

## Abstract

In general, only one diffusion model would be applied to whole field-of-view voxels in the intravoxel incoherent motion-magnetic resonance imaging (IVIM-MRI) study. However, the choice of the applied diffusion model can significantly influence the estimated diffusion parameters. The quality of the diffusion analysis can influence the reliability of the perfusion analysis. This study proposed an optimal model mapping method to improve the reliability of the perfusion parameter estimation in the IVIM study. Six healthy volunteers (five males and one female; average age of 38.3 ± 7.5 years). Volunteers were examined using a 3.0 Tesla scanner. IVIM-MRI of the brain was applied at 17 b-values ranging from 0 to 2,500 s/mm^2^. The Gaussian model, the Kurtosis model, and the Gamma model were found to be optimal for the CSF, white matter (WM), and gray matter (GM), respectively. In the mean perfusion fraction (f_p_) analysis, the GM/WM ratios were 1.16 (Gaussian model), 1.80 (Kurtosis model), 1.94 (Gamma model), and 1.54 (Optimal model mapping); in the mean pseudo diffusion coefficient (D^*^) analysis, the GM/WM ratios were 1.18 (Gaussian model), 1.19 (Kurtosis model), 1.56 (Gamma model), and 1.24 (Optimal model mapping). With the optimal model mapping method, the estimated f_p_ and D^*^ were reliable compared with the conventional methods. In addition, the optimal model maps, the associated products of this method, may provide additional information for clinical diagnosis.

## Introduction

The theory of intravoxel incoherent motion (IVIM) was first introduced to extend the understanding and usefulness of diffusion-weighted imaging (DWI) with the motion probing gradients (MPG) in the mid-1980s (Le Bihan et al., 1986, 1988). The potential of IVIM-MRI to simultaneously determine perfusion and diffusion information has led to high expectations regarding clinical applications (Le Bihan and Turner, 1992; Paschoal et al., 2018). However, the feasibility of perfusion measurement with IVIM-MRI has been controversial for a long time. One of the reasons is the instrumental limitations in the past. It has been challenging to estimate small blood volumes with a low signal-to-noise ratio (SNR) in IVIM-MRI. In addition, numerous perfusion MRI techniques have been well-developed in the last three decades; e.g., dynamic susceptibility contrast MRI, dynamic contrast-enhanced MRI, and arterial spin labeling (Alsop et al., 2015; Welker et al., 2015; Zhang et al., 2017). IVIM-MRI, especially in the brain, has not been thought to be practically feasible for performing perfusion measurements.

In the last decade, the improvements in MRI instrumentation, such as high SNR echo-planar imaging, have increased interest in IVIM-MRI, which is anticipated to be an alternative technique for non-invasive perfusion measurement. Based on the theoretical relationship, the perfusion fraction (*f*_*p*_) and pseudo-diffusion coefficient (D^*^) obtained by IVIM-MRI can represent the cerebral blood volume (CBV) and inverse mean transit time (MTT^−1^) of classic perfusion, respectively (Le Bihan and Turner, 1992). Numerous clinical studies have demonstrated the applications of IVIM-MRI to cancer, stroke, and Moyamoya disease in the brain (Puig et al., 2016; Yao et al., 2016; Li et al., 2018; Federau et al., 2019).

Although some previous studies have demonstrated a correlation between *f*_*p*_ and CBV by conventional perfusion MRI (Wirestam et al., 2001; Federau et al., 2014a,b), others have shown controdictory results (Wu et al., 2015; Puig et al., 2016; Hara et al., 2019). Additionally, arguments for a relationship between D^*^ and MTT^−1^ have been made (Wirestam et al., 2001; Federau et al., 2014b; Wu et al., 2015; Hara et al., 2019). Furthermore, in conventional perfusion studies, the CBV ratios of gray matter (GM) and white matter (WM) have been found to range from 1.4 to 1.8, and the MTT^−1^ ratio of GM and WM has ranged from 1.2 to 1.5 (Greenberg et al., 1978; Leenders et al., 1990; Wirestam et al., 2001; Shin et al., 2007; Carroll et al., 2008). However, relatively higher *f*_*p*_ ratios (~2) of GM and WM and lower D^*^ ratios (~ ≤ 1) of GM and WM have been commonly estimated in IVIM studies (Wirestam et al., 2001; Federau et al., 2014b; Wu et al., 2015; Bertleff et al., 2017).

Notably, the quality of the diffusion analysis can influence the reliability of the perfusion analysis. In general, only one diffusion model would be applied to the voxels in the whole field-of-view in an IVIM-MRI study. However, the choice of the applied diffusion model can significantly influence the perfusion estimation (Lu et al., 2012; Pavilla et al., 2017). Because of the heterogeneity of tissue structures, assigning a single diffusion model to the whole brain might induce systematic errors in the perfusion estimation. The study aim was to determine the optimal diffusion models in brain IVIM-MRI. In addition, we propose an optimal model mapping method to improve the reliability of perfusion parameter estimation in IVIM studies.

## Materials and Methods

### Theory

The signal intensity of IVIM-MRI is regarded as a two-compartment model by Le Bihan et al. (1988):

(1) $$\begin{array}{r} S\left(b\right)=S_{e0}\cdot E\left(b;D,K\right)+S_{v0}\cdot \text{exp}\left(-bD^{*}\right) \end{array}$$

where *S*(*b*) denotes the signal intensity depending on *b*-values, *S*_*v*0_ and *S*_*e*0_ are the non-MPG-induced signal intensities of the intravascular and extravascular components, respectively; *E*(*b; D, K*) is the attenuation function of the extravascular component, i.e., the diffusion model, with two parameters *D* and *K* that denote the diffusion coefficient and diffusional Kurtosis, respectively; and D^*^ is the pseudo-diffusion coefficient, which indicates the blood random microcirculation and is considered to relate MTT as mentioned.

The perfusion fraction, i.e., the volume fraction of flowing blood, has been defined by Jerome et al. (2016):

(2) $$\begin{array}{r} f_{p}=\frac{S_{v0}}{S_{v0}+S_{e0}} \end{array}$$

Note that *f*_*p*_ and D^*^, crucial parameters in IVIM, are challenging to evaluate with sufficiently high reliability because of the negligible contribution of the perfusion component to the total MPG-induced signal [the second term in Equation (1)]. Therefore, a precise estimation of the diffusion-weighted component, the first term in Equation (1), is essential.

**Figure 1:**
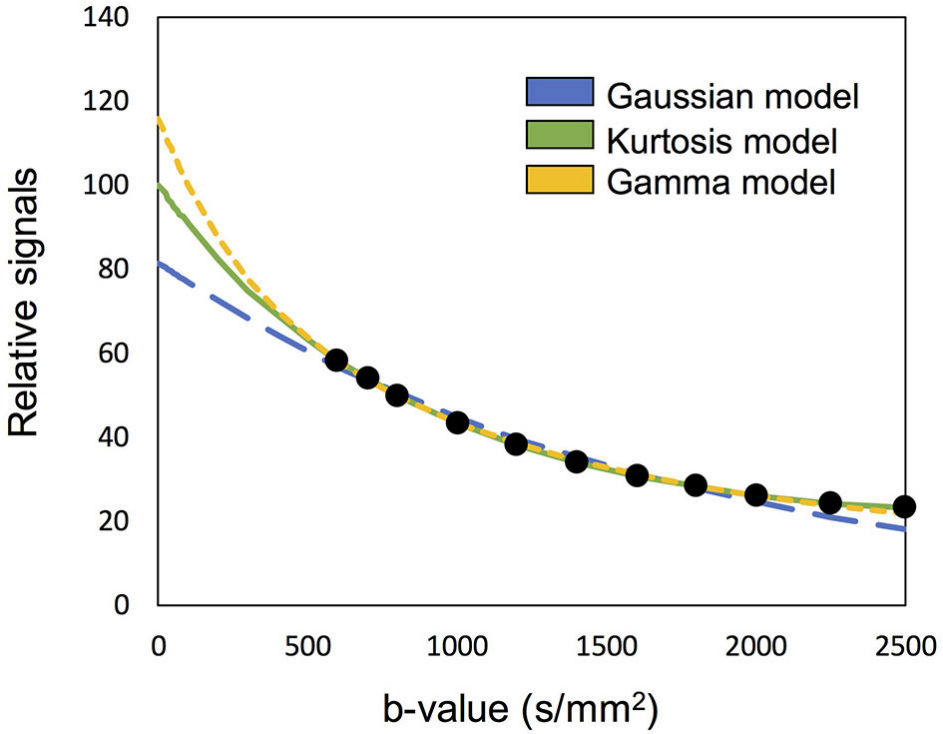
Demonstration of the high b-valued IVIM-MRI data fitted with three different diffusion models. The estimated Se0 are varied to influence the fp estimation.

The concept of “optimal model mapping” is to apply an appropriate diffusion model, *E*(*b; D, K*), to the examined voxel based on the goodness-of-fit and not *a priori* tissue information. In this study, three types of diffusion models, namely Gaussian, Kurtosis, and Gamma models, were chosen to be the candidates of *E*(*b*), as described in the later sections. Figure 1 which shows an example of IVIM-MRI data for high b-values that are fitted with three different diffusion models, demonstrates that the estimated *S*_*e*0_ values are varied by the applied models. To determine the optimal model for an individual voxel, the corrected Akaike Information Criterion (cAIC) (Akaike, 1987; Hurvich and Tsai, 1989) was applied to illustrate the goodness-of-fit of each candidate model. The cAIC is calculated according to the following equation:

(3) $$\begin{array}{r} cAIC=2P+n\;ln\left(\frac{RSS}{n}\right)+\frac{2P\left(P+1\right)}{n-P-1} \end{array}$$

where *n* is the number of data points, *P* is the number of parameters, and *RSS* is the residual sum of squares. A diffusion model with the minimal cAIC within the candidates was considered to be optimal for the examined voxel.

Numerous physical and mathematical models were presented in the diffusion MRI history (Jensen and Helpern, 2010). To represent the tissue structure as well as to minimize the number of the parameters of the model to estimate, three typical diffusion models were selected in this study.

#### Gaussian Model

For individual molecules of free water, the Gaussian distribution probability displacement along one direction is considered. Then, the attenuation function can be derived, as shown by Le Bihan et al. (1986):

(4) $$\begin{array}{r} E\left(b\right)=exp\left(-bD\right) \end{array}$$

where *D* is the diffusion coefficient. In the brain, the ventricles are filled with cerebrospinal fluid (CSF). Because CSF is uniform and the ventricles are large cavities, the Gaussian model was appropriate for the diffusion analysis.

In brain tissues, e.g., GM and WM, water molecules can interact with cell membranes and microstructures, so the displacement probability of water diffusion might not show a Gaussian distribution. That is, a non-Gaussian model is needed to evaluate restricted water diffusion.

#### Kurtosis Model

To describe the deviation from a Gaussian distribution, a mathematical model was proposed by Jensen et al. (2005):

(5) $$\begin{array}{r} E\left(b\right)=\exp\left(-bD+\frac{b^{2}D^{2}K}{6}+O\left(b^{3}\right)\right) \end{array}$$

where *K* is the diffusional Kurtosis, a dimensionless statistical metric to quantify the non-Gaussian characteristic of an arbitrary probability distribution (DeCarlo, 1997), and *O*(*b*^3^) is the power series of the higher-order terms. When *O*(*b*^3^) is considered to be negligible, this model can be rewritten as follows:

(6) $$\begin{array}{r} E\left(b\right)=\exp\left(-bD+\frac{b^{2}D^{2}K}{6}\right) \end{array}$$

where when *K* = 0, Equation (6) reduces to Equation (4) as a Gaussian model. In the Kurtosis model, the maximum b-value should be within an adequate range, which is determined by the given D and K; it should be sufficiently high to express the non-Gaussian effect and sufficiently small to neglect the higher-ordered exponential terms (Jensen and Helpern, 2010). However, there is no way to determine the boundary of the maximum *b*-value without the information of D and K. In practice, assuming a monotonically decreasing *S*(*b*), the upper bound *b* ≤ 3/*DK* for applied *b*-values should be considered (Jensen et al., 2005).

#### Gamma Model

To illustrate a tissue structure with a high number of compartments, we can consider a statistical model based on a Gamma distribution of diffusion coefficients (Jensen and Helpern, 2010; Oshio et al., 2014). The fraction density function **ρ** (*D'*) for a compartment is given by:

(7) $$\begin{array}{r} \rho \left(D^{\prime }\right)=\frac{\beta^{\alpha }}{\Gamma \left(\alpha \right)}{\left(D^{\prime }\right)}^{\alpha -1}\exp\left(-bD^{\prime }\right) \end{array}$$

where *D*′ is the diffusion coefficient in the compartment, and the mean and variance of the Gamma distribution are given by α/β and α/β^2^, respectively. Then, the decay curve E(*b*) is given by:

(8) $$\begin{array}{r} E\left(b\right)=\int_{0}^{\infty }\rho \left(D^{\prime }\right)\exp\left(-bD^{\prime }\right)=\frac{\beta^{\alpha }}{{\left(\beta +b\right)}^{\alpha }} \end{array}$$

According to Jensen's work, α and β can be given by comparison of the Taylor expansion of Equation (6) with Equation (5), as α = *3/K* and β = *3/KD*. Then, Equation (7) can be rewritten using D and K as:

(9) $$\begin{array}{r} E\left(b\right)={\left(1+\frac{bDK}{3}\right)}^{-\frac{3}{K}} \end{array}$$

Here, we should note that D coincides with the expected value of D′ of the Gamma distribution and that K satisfies the assumption of a multiple-compartment model without water exchange (Jensen et al., 2005):

(10) $$\begin{array}{r} K=\frac{3\sigma^{2}}{D^{2}} \end{array}$$

Like the Kurtosis model, this model also converges to the Gaussian model when *K* = 0. Likewise, if the applied *b-*value is sufficiently smaller than 27/(6*DK*), the third-order or higher terms of the Taylor expansion of Equation (9) are negligible, resulting in the coincidence of the two models (Jensen and Helpern, 2010). Conversely, considering that typically *DK* is around 0.001 mm^2^/s, b-values smaller than 600 s/mm^2^, which provides <1% signal difference, can be insufficient to distinguish the two models.

### MRI Acquisition

This study was approved by the Ethics Committee Graduate School and Faculty of Medicine Kyoto University. All participants provided written informed consent. Six healthy volunteers (five males and one female; average age: 38.3 ± 7.5 years) were scanned by using a 3.0T scanner (MAGNETOM Trio Tim; Siemens Healthineers, Erlangen, Germany) with a 32-channel phase-array head coil. The multiband DWI sequence provided by the University of Minnesota was used for imaging (Feinberg et al., 2010; Moeller et al., 2010; Xu et al., 2013), but no multiband or parallel imaging function was adopted to avoid a non-uniform SNR distribution. IVIM-MRI was performed with spin-echo echo-planar imaging and MPGs. Seventeen *b*-values of 0, 100, 200, 300, 400, 500, 600, 700, 800, 1,000, 1,200, 1,400, 1,600, 1,800, 2,000, 2,250, and 2,500 s/mm^2^ in six MPG directions ([1,1,0], [0,1,1], [1,0,1], [1,−1,0], [0,1,−1], [−1,0,1]) were used. The other imaging parameters were TR/TE = 2,600/80 ms; flip angle = 90°; voxel size = 3 × 3 × 3 mm^3^; acquisition matrix size = 64 × 48 (3/4 partial Fourier); image matrix size = 64 × 64; 22 slices and slice gap = 3 mm. To assess the influence of SNR difference on the selection of the optimal model, the IVIM-MRI acquisition was repeated six times. A three-dimensional T1-weighted gradient-echo sequence (magnetization-prepared rapid gradient-echo; MP-RAGE) was acquired to obtain anatomical information. The acquisition parameters for the MP-RAGE scan were TR/TE/TI = 2,500/3.4/990 ms; flip angle = 8°; voxel size = 0.9375 × 0.9375 × 0.9333 mm^3^; image matrix size = 256 × 256 × 192. The acquisition time for a single IVIM-MRI set was 6 min 48 s in our study.

### Data Analysis

All data analysis was performed by using Matlab (R2013a; The MathWorks Inc., Natick, MA). The head movement was confirmed by using Statistical Parametric Mapping (SPM 12; Wellcome Centre for Human Neuroimaging, University College London, London, England). All data showed lower than 3 mm (one voxel size) movement. To assess the influence of SNR on the data analysis, six datasets with different SNR were generated by changing the number of averages from one to six. They were named as NA1–NA6, where “NA” denotes the number of averages.

To account for the effect of Rician noise in the fitting, the measured signal (Ŝ) was modeled as:

(11) $$\begin{array}{r} {\hat{S}}^{2}\left(b\right)=S^{2}\left(b\right)+NCF \end{array}$$

where *S* denotes the noise-free signal intensity defined in Equation (1) and *NCF* is the noise correction factor to characterizes the “intrinsic” noise contribution (Iima et al., 2015). In this study, *NCF* was estimated from the square of the mode value of the signal histogram of the *S*(0) image.

Two-step asymptotic curve fitting (Pekar et al., 1992) was performed voxel-by-voxel with the IVIM-MRI series for each subject. The optimal model maps were obtained within the diffusion analysis.

At the first step of the asymptotic fitting, only high *b*-value IVIM-MRI data (*b* = 600–2,500 s/mm^2^) were used to minimize the signal contamination from the intravascular component and to simplify the IVIM model assigned by Equation (1) by neglecting its perfusion term.

(12) $$\begin{array}{r} S\left(b\right)\approx S_{e0}\cdot E\left(b\right) \end{array}$$

**Figure 2:**
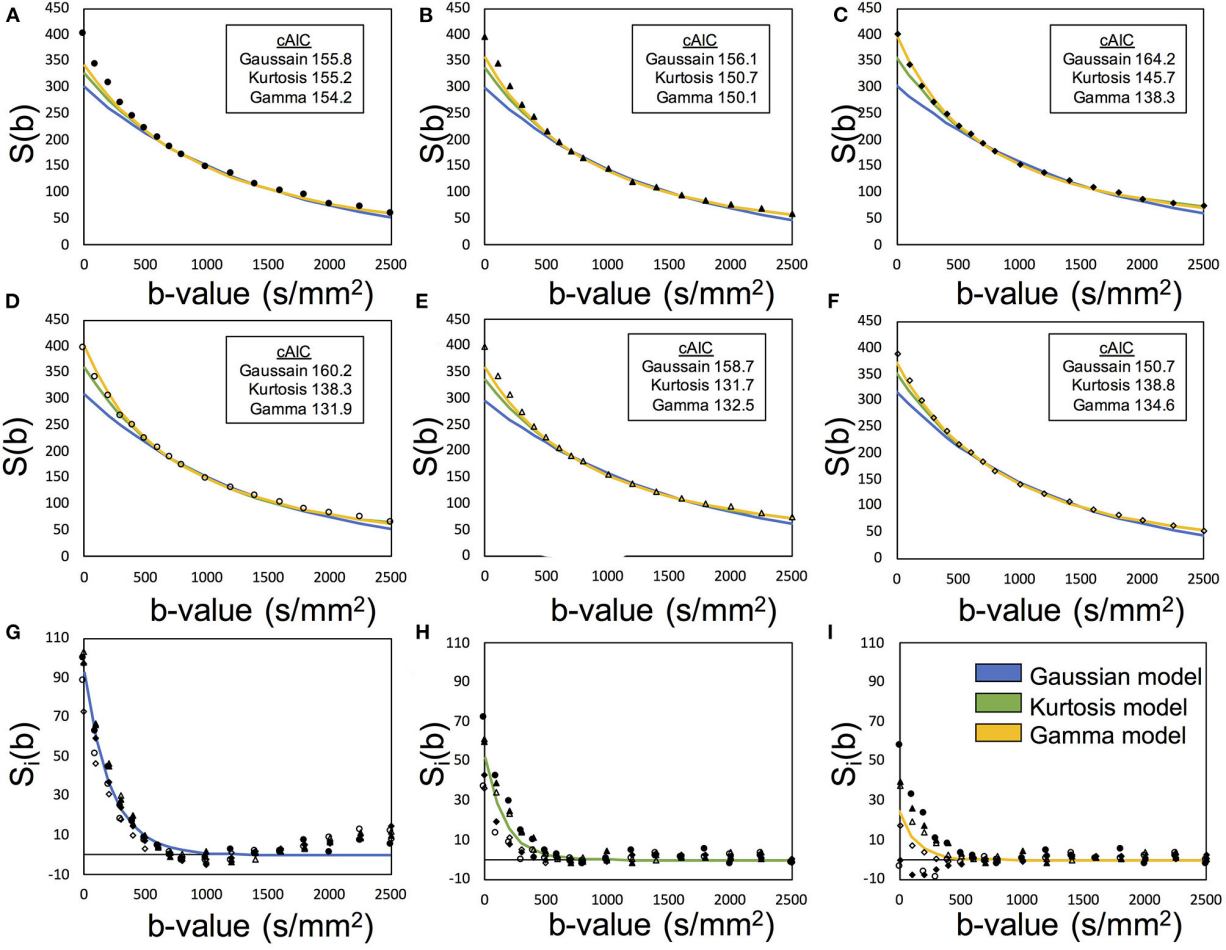
Demonstration of the two-step asymptotic fitting of a gray matter voxel. The blue, green, and yellow curves indicated the fittings by Gaussian model, Kurtosis model, and Gamma model, respectively. **(A–F)** showed the diffusion fitting along six MPG directions. **(G–I)** showed the residual signals by removing diffusion components with Gaussian model fitting, Kurtosis model fitting, and Gamma model fitting. The mark styles of solid circle, solid triangle, solid square, hollow circle, hollow triangle, and hollow square indicated the data from the MPG directions of [1,1,0], [0,1,1], [1,0,1], [1,-1,0], [0,1,-1], and [−1,0,1], respectively.

Figure 2 presents an example of the IVIM analysis. Six series with varied MPG directions were independently analyzed. Data fits were performed by using Equation (12) associated with the candidate model (Equations 4, 5, and 8) (Figures 2A–F). Then, six sets of the diffusion-related parameters (*S*_*e*0_, *D*, and *K*) were estimated. For the *f*_*p*_ estimation later, six estimated *S*_*e*0_ values were averaged. The mean diffusibility (*MD*) was calculated by averaging the results of six MPG directions (Pierpaoli et al., 1996). Because of insufficient Kurtosis tensors in this study, we could not estimate the mean Kurtosis. Alternatively, the apparent mean Kurtosis (${\bar{\text{K}}}_{\text{app}}$) was calculated by averaging the results of six MPG directions for comparison of the diffusion models.

In the perfusion analysis in the second step of the asymptotic fitting, six series of the intravascular component were extracted by removing the extravascular component from the *S*(*b*) series (Equation 1). Assuming an isotropic capillary perfusion, the six series were simultaneously fitted by:

(13) $$\begin{array}{r} {Ŝ}_{v,i}\left(b\right)=S_{v0}\cdot \exp\left(-bD^{*}\right) \end{array}$$

where Ŝ_*v, i*_ was the extracted perfusion series and suffix *i* denotes the MPG direction. Then, *f*_*p*_ was estimated with *S*_*v*0_ and the averaged *S*_*e*0_with Equation (2). With these processed, one set of the perfusion-related parameters (*f*_*p*_ and D^*^) were finally estimated (Figures 2G–I).

The optimal model was defined as the model that yielded the minimal mean cAIC of the diffusion estimation. To evaluate the optimal model for the individual voxels, six cAICs by MPG directions of each candidate model were calculated by Equation (3). An optimal model map was generated to display the optimal model for each voxel. Then, the hybrid modeling maps of *f*_*p*_, D^*^, *MD*, and ${\bar{K}}_{app}$ were generated by assigning the optimal results to the whole brain matrix.

### Validation With Regional Analysis

To determine the relationship between the optimal model and corresponding tissue, representative values of the parameters estimated with our method were calculated for each of the GM/WM/CSF regions. Each region was decided by segmenting the MP-RAGE images by using SPM 12 and the segmented volumes were co-registered to the *b*_0_ images. To avoid the errors, such as from the partial volume effect, mis-segmentation, mis-registration, and image distortion on IVIM images, the segmented voxels of an identical proportion >95% were collected. In addition, in the tissue regions (GM and WM), the voxels of *MD* > D^*^ were excluded, whereas in the CSF region, the voxels of ${\bar{K}}_{app}$ > 0.1 were excluded. After the determination of regions, the mean value of each parameter obtained with the hybrid modeling method was calculated for each subject as a representative value.

## Results

**Figure 3:**
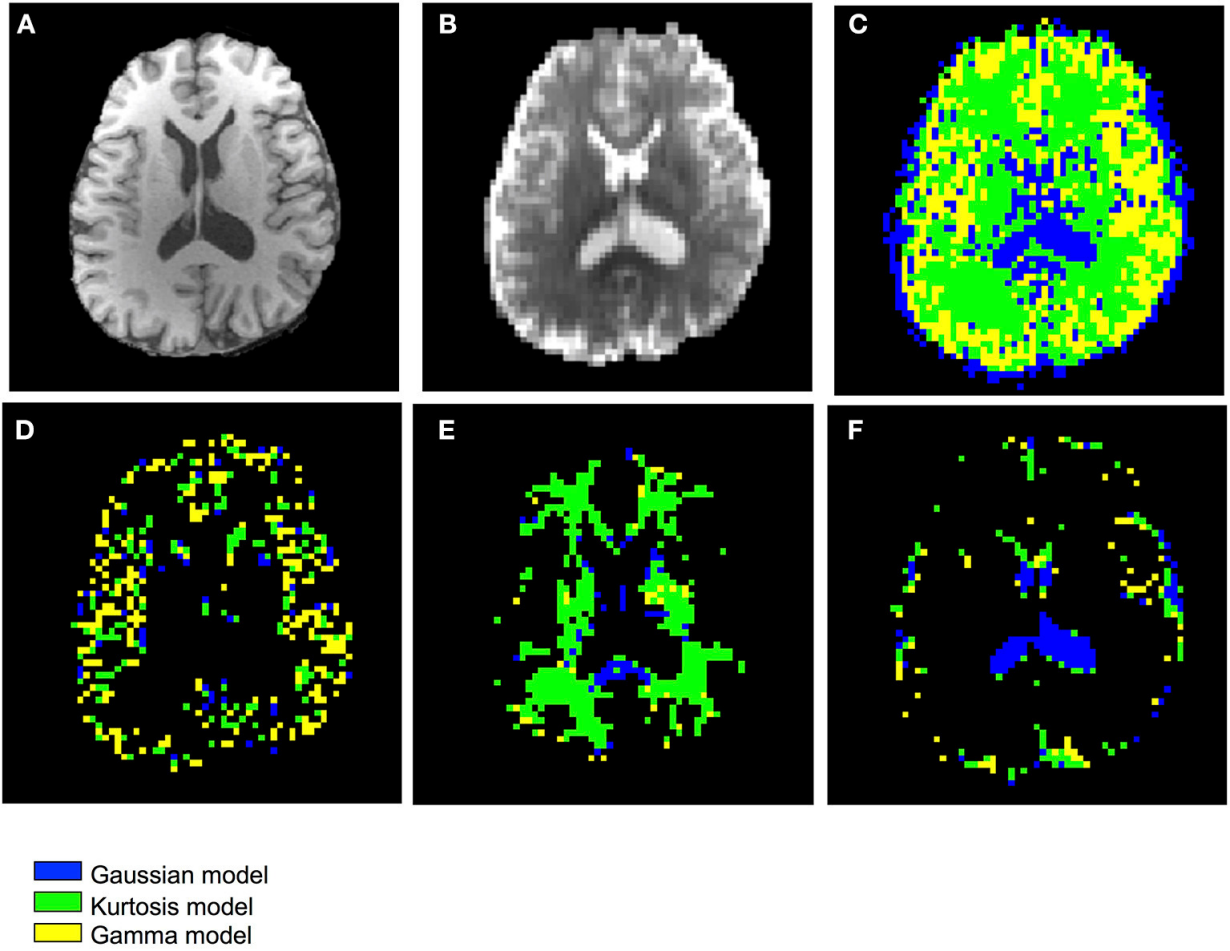
Demonstration of the anatomical maps and the optimal model maps: **(A)** T1-weighted (MP-RAGE) image **(B)** b_0_ image of the IVIM-MRI series **(C)** optimal model map **(D)** optimal model map of 95% GM **(E)** optimal model map of 95% WM **(F)** optimal model map of 95% CSF.

Figure 3 shows the T1-weighted image by MP-RAGE (A), the b_0_ image (B), the full optimal model map (C), and the segmented map by GM (D) WM (E), and CSF (F) of one subject with the NA6 data. The optimal model maps of six subjects by varied NA was shown in the Supplementary Material. Note that the segmented optimal model map for CSF (Figure 3F) contained the voxels of K ≥ 0.1. Table 1 presents the mean proportional size of each optimal model in each region of the six subjects. In general, GM was Gamma model dominant (45.3%), WM was Kurtosis model dominant (80.9%), and CSF (K < 0.1) was Gaussian model dominant (100%).

**Table 1:** Proportional territory of the optimal models in each region (%).

	**GM**	**WM**	**CSF**
Gaussian	18.7 ± 3.0	9.9 ± 1.8	100 ± 0.0
Kurtosis	36.1 ± 1.9	80.9 ± 1.9	N/A
Gamma	45.3 ± 3.8	9.3 ± 3.1	N/A

**Figure 4:**
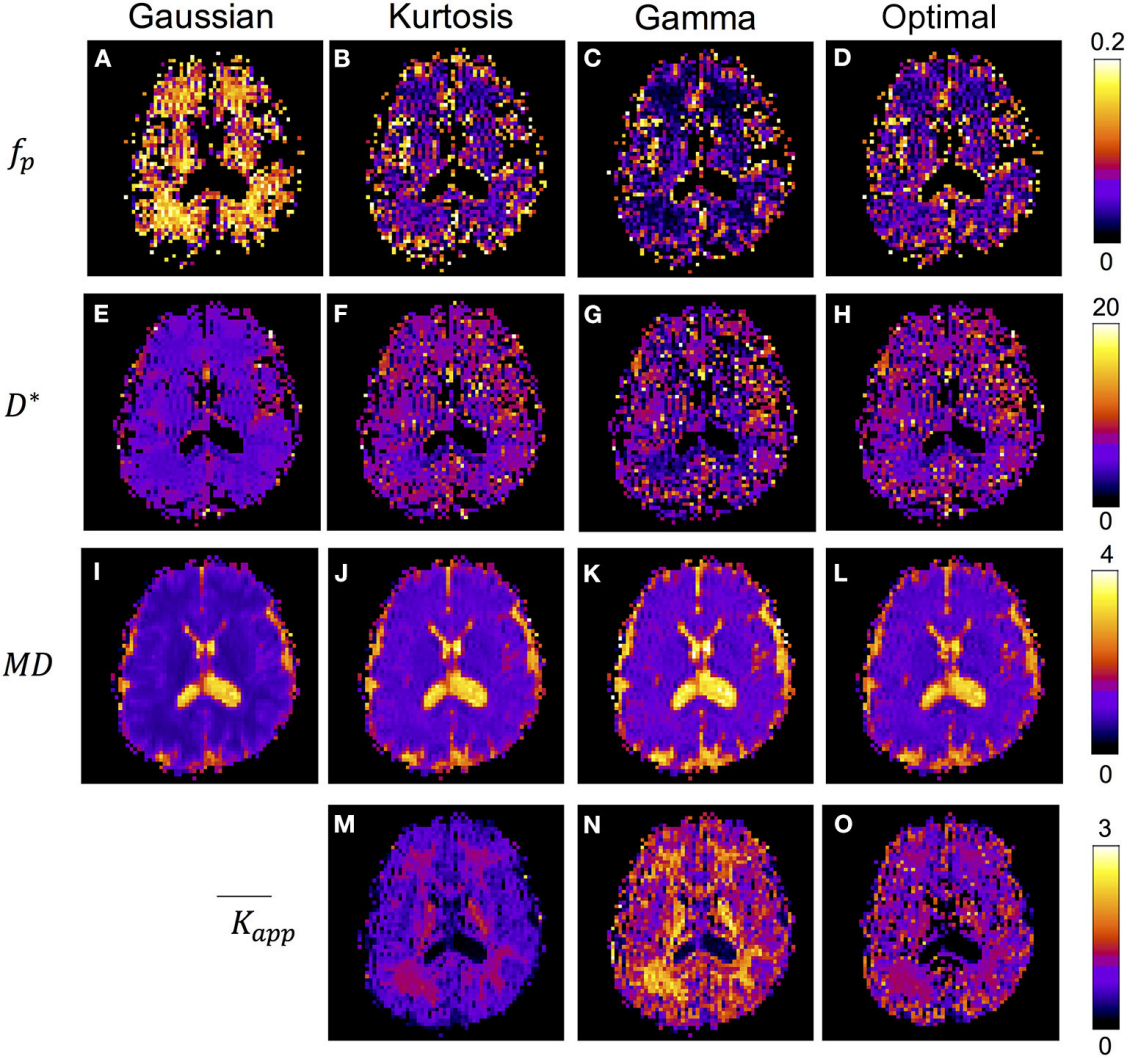
IVIM parameter maps respectively analyzed by Gaussian model **(A,E,I)**, Kurtosis model **(B,F,J,M)**, Gamma model **(C,G,K,N)**, and the optimal model mapping **(D,H,L,O)** for one subject. The units for f_*p*_ is % and for D* and MD are ×10^−3^ mm^2^/s.

**Figure 5:**
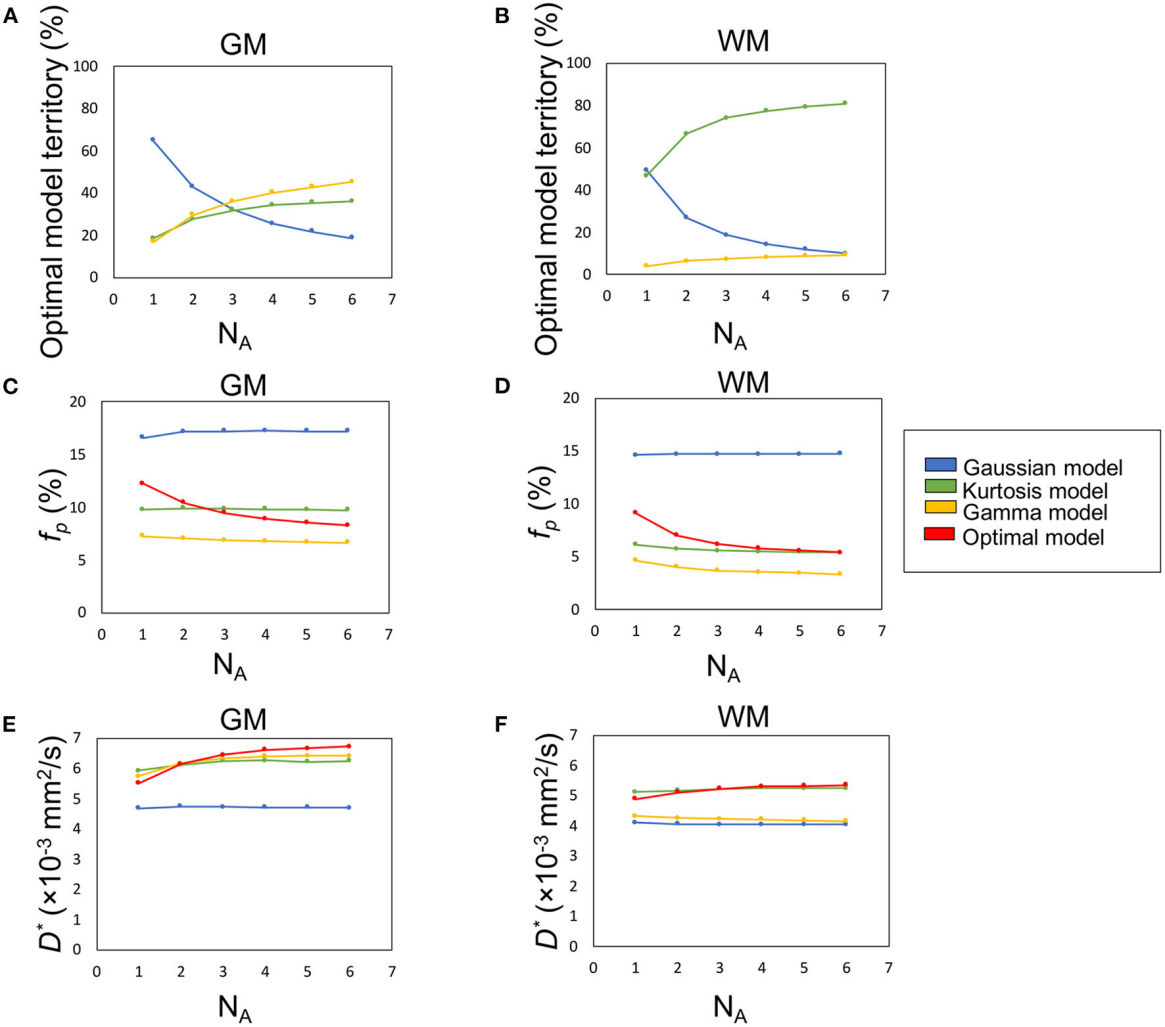
The influence of SNR (denoted by number of averages, NA) on the percentage optimal model territory **(A,B)**, *f*_*p*_ **(C,D)**, and D^*^ **(E,F)**.

Figure 4 shows the IVIM parameter maps obtained by the conventional method with only the three candidate diffusion models and the proposed method. Note that in the *f*_*p*_ and D^*^ maps, the CSF region was masked to enhance the GM/WM contrast. In the *f*_*p*_ maps, results of the Gaussian model (Figure 4A) showed several times higher values than those of the other two models (Figures 4B,C). In the D^*^ maps, the GM values were generally higher than the WM values. The results estimated by the Gaussian model (Figure 4E) were lower than those of the other two models (Figures 4F,G). In the MD maps, the CSF regions showed similar results in all methods. The MD map of Gaussian model (Figure 4I) showed similar GM/WM contrast and lower absolute values when compared with the other three methods (Figures 4J–L). In the ${\bar{K}}_{app}$ maps, both the Kurtosis model (Figure 5M) and Gamma model (Figure 5N) showed higher ${\bar{K}}_{app}$ values in WM than in GM. However, with the optimal model mapping method (Figure 5O), the ${\bar{K}}_{app}$ differences between GM and WM were small. Table 2 summarizes the IVIM parameters of each tissue region according to the models. In the mean *f*_*p*_ analysis, the GM/WM ratios were 1.16 (Gaussian model), 1.80 (Kurtosis model), 1.94 (Gamma model), and 1.54 (Optimal model mapping); in the mean D^*^ analysis, the GM/WM ratios were 1.18 (Gaussian model), 1.19 (Kurtosis model), 1.56 (Gamma model), and 1.24 (Optimal model mapping).

**Table 2:** IVIM parameter means of six subjects (Mean ± standard deviation).

	***f**_**p**_* **(%)**		D^*^ **(×10**^****−3****^ **mm**^****2****^**/s)**		**MD (× ** **10**^****−3****^ **mm**^****2****^**/s)**			**MK**_**app**_	
	**GM**	**WM**	**GM**	**WM**	**GM**	**WM**	**CSF**	**GM**	**WM**
Gaussian	17.2 ± 0.8	14.8 ± 0.2	4.7 ± 0.1	4.0 ± 0.0	0.72 ± 0.02	0.58 ± 0.01	2.91 ± 0.12	N/A	N/A
Kurtosis	9.7 ± 0.7	5.4 ± 0.4	6.2 ± 0.2	5.2 ± 0.1	0.90 ± 0.03	0.79 ± 0.02	2.94 ± 0.12	0.55 ± 0.01	0.82 ± 0.03
Gamma	6.6 ± 0.6	3.4 ± 0.3	6.4 ± 0.3	4.1 ± 0.2	1.05 ± 0.05	0.89 ± 0.02	3.01 ± 0.13	1.02 ± 0.02	1.55 ± 0.05
Optimal	8.3 ± 0.6	5.4 ± 0.3	6.7 ± 0.2	5.4 ± 0.1	0.97 ± 0.04	0.79 ± 0.02	2.91 ± 0.12	0.77 ± 0.02	0.85 ± 0.02

Table 3 presents the regional results of the Kurtosis model and Gamma model. In the mean *f*_*p*_ analysis, the Kurtosis model yielded higher *f*_*p*_ estimation in the voxels for which the optimal model was the Gamma model; the Gamma model yielded lower *f*_*p*_ estimation in the voxels for which the optimal model was the Kurtosis model. Figures 1, 2 also showed the intrinsic mathematical property of these two models. In the WM, the *f*_*p*_ estimations were similar to the optimal results. Similarly, in the mean D^*^ analysis, the Kurtosis model yielded higher D^*^estimation in the voxels for which the optimal model was the Gamma model; the Gamma model yielded lower D^*^estimation in the voxels for which the optimal model was the Kurtosis model.

**Table 3:** Regional analysis with a single model. Pool-K or Pool-G indicated that the optimal model for the voxel clusters were Kurtosis model or Gamma model, respectively. For example, in the Pool-K of GM, the mean *f*_*p*_ was 6.1% by Kurtosis model and 3.8% by Gamma model.

	***f***_***p***_ **(%)**				D^*^ **(× ** **10**^****−3****^ **mm**^****2****^**/s)**			
	**GM**		**WM**		**GM**		**WM**	
	**Pool-K**	**Pool-G**	**Pool-K**	**Pool-G**	**Pool-K**	**Pool-G**	**Pool-K**	**Pool-G**
Kurtosis	6.1 ± 0.6	13.3 ± 0.7	4.5 ± 0.4	10.0 ± 0.5	6.5 ± 0.2	6.5 ± 0.1	5.3 ± 0.2	6.0 ± 0.1
Gamma	3.8 ± 0.5	8.7 ± 0.6	2.6 ± 0.4	5.9 ± 0.6	5.4 ± 0.3	7.9 ± 0.3	3.8 ± 0.2	7.5 ± 0.4

### Effect of SNR

Figures 5A,B demonstrate the optimal model territories by different NA in GM and WM, respectively. On increasing NA, the Gamma and Kurtosis models became more dominant in GM and WM, respectively. In contrast, the Gaussian model showed a decline. In addition, there were changes in estimated *f*_*p*_ (Figures 5C,D) and D^*^ (Figures 5E,F) on increasing NA in the parenchymal regions. All curves achieved plateaus when NA was larger than five.

**Figure 6:**
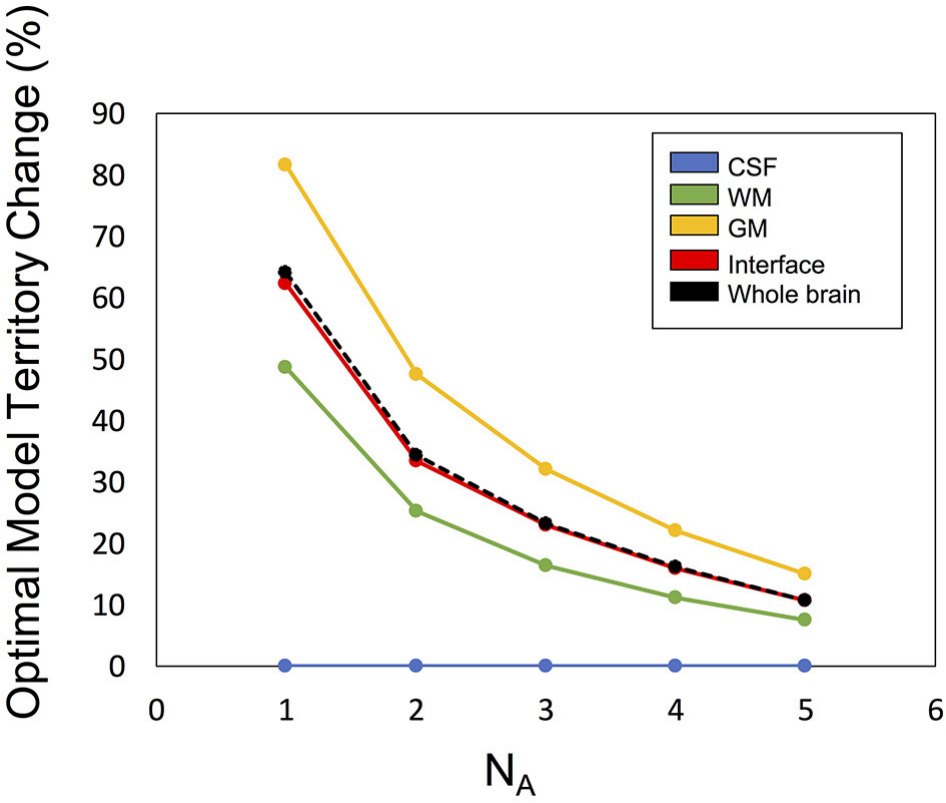
The optimal model territory change by SNR (denoted by number of averages, NA) compared with the NA6 dataset.

Figure 6 illustrates the optimal model territory changes when compared with the NA6 data. In the whole brain, the optimal model switch happened in 64.2% (NA1), 34.3% (NA2), 23.2% (NA3), 16.0% (NA4), and 10.8% (NA5) voxels.

## Discussion

In this study, we described an optimal model mapping method to improve the quantification uncertainty due to the heterogeneity of tissue structures. We applied this method to six normal subjects and found that the inter-subject variability was very small. In addition, the optimal model map showed tissue-specific patterns without *a priori* tissue information. The method results indicate that reliable and reproducible perfusion-related parameters, i.e., *f*_*p*_ and D^*^, could be estimated successfully by using IVIM-MRI. This method might have potential to identify additional physiological biomarkers for clinical diagnosis.

About the availability to apply the Kurtosis model and the Gamma model to the diffusion analysis, two criteria were mentioned in the theory part. On the first criterion, b ≤ 3/DK for the Kurtosis model, Table 2 showed the products of MD and ${\bar{K}}_{app}$ are about 0.50 (GM) and 0.65 μm^2^/ms (WM), respectively, so that maximum b-value (2,500 s/mm^2^) is satisfied at the upper limit of 6,000 s/mm^2^ (GM) and 4,620 s/mm^2^ (WM). In the same way, on the second criterion, b ≪ 27/(6DK) for the Gamma model, the products are about 1.07 (GM) and 1.38 (WM), respectively, so that, when the applied b-values is sufficiently smaller than 4,200 s/mm^2^ (GM) or 3,260 s/mm^2^, the curves by Kurtosis model and Gamma model become similar. This suggested that our maximum b-value of 2,500 s/mm^2^ was sufficiently high to see the difference between the two models.

To express the non-Gaussian diffusion, the Kurtosis and Gamma models were selected as the candidates. However, we did not adopt the biexponential model which is commonly used because of the difference in the number of associated parameters (three for the former two models and four for the latter) (Kiselev and Il'yasov, 2007). The concept of AIC is that a model with a larger number of parameters will fit well so that the resultant RSS is smaller, as shown in Equation (3). For example, consider a comparison of three models with different numbers of parameters (*p* = 2, 3, and 4) but with the same number of points to fit (*n* = 11). In this case, to obtain the same cAICs, the RSSs of the three- and four-parameter models must be 70.0 and 43.5%, respectively, of that of the two-parameter model. Considering that RSSs are sufficiently small in case of Kurtosis and Gamma models, an additional parameter in the biexponential model can be critical. Indeed, when we added the biexponential model to the candidates, it became the optimal model at very few numbers of voxels in the tissue boundary region (not shown here). In addition, this tendency is depicted in Figure 5, where the Gaussian model (two-parameter model) was dominant in GM when the SNR was low, but the optimal model switched gradually to Gamma with SNR increase.

The optimal model maps (Figure 3C) showed strong correlation between the applied diffusion model and tissue type. The Gaussian model, which presents free water in a single compartment, dominated the ventricle regions (CSF), a water pool. The Kurtosis model, which presents restricted water with a non-Gaussian probability distribution movement whose decay curve can be approximated with the Equation (6), dominated WM regions containing axon fibers. The Gamma model, which presents a multiple-compartment tissue with statistical distribution on diffusion coefficients, dominated GM regions containing neuronal cell bodies. Having said that, not all of the GM voxels showed Gamma model optimum and some WM voxels showed non-Kurtosis model optimum (Table 1). For example, Figure 3 showed that in the thalamus and basal ganglia, the optimal model indices were varied and dominated by the Kurtosis model. Owing to the optimal model territory achieving steady state (Figure 5) with NA6 dataset, the influence of the noise was expected to be relatively small. Some reasons might cause the complex optimal model contents. First, he model of water movement can be varied in a type of tissue. Although most of the voxels followed a specific model, others did not. Table 3 demonstrates that an inadequate diffusion model fitting can propagate systematic errors to the perfusion estimation. Second, in this study, the voxel size was 3 × 3 × 3 mm^3^, which is close to the GM thickness. Although we segmented the regions of interest with the MPRAGE volume by SPM, the mis-registration caused by the DWI distortion can be happened. The voxels located around the tissue boundaries might be replaced by other tissues. Third, based on the tendency of Figure 5, the Gaussian voxels might shift to the Gamma voxels or the Kurtosis voxels by increasing SNR. On the other hand, despite it is possible to apply the optimal model mapping method based on the segmental regions of interest for the normal subjects (Table 2), in clinical use, we would not expect the tissue type a priori, especially for the abnormal legions. These issues need further investigations.

The cAIC values in Figure 2 may give the impression that their small differences among models indicate the selection of the optimal model just by chance, being affected by noise. Nevertheless, we should be careful about the evaluation regardless of however small the differences might be. We should not refer to their absolute values for comparison since scaling the original data can shift them even though the final results remain unchanged. As shown in the definition of cAIC (Equation 3), when the original data are scaled up or down by the factor of alpha, the cAIC values shift by 2N × ln (alpha). Therefore, if we scale by a factor of 1/1,000, for example, the cAIC values will shift by −138.2. Thus, in the case of Figure 2F, the cAIC values (150.7, 138.8, and 134.6) for the Gaussian, Kurtosis, and Gamma models will shift to 12.5, 0.6, and −3.6, respectively, which would give a different impression. Here, we should emphasize that this scaling does not change the entire results, including *f*_*p*_, D, D^*^, and optimal models.

The most convincing way to evaluate the significance of the cAIC differences is to compare the differences statistically with the variation of each cAIC value in iterative trials. For example, by acquiring 6N datasets (i.e., N sets of six-average data), we can get a set of N cAIC values for each model at a voxel. Thereafter, we can evaluate whether the difference between the cAICs of two models is significant by applying the paired *t*-test to the two sets of N cAIC values.

Considering that the total scan time extends by N times, this approach is not practical. However, there are three tendencies strongly suggesting that our method does not select the optimal model by chance. First, the model maps show the voxel clusters of each model. If the model selection is affected by noise, the model map would be noisier and exhibit a random pattern. Second, the monotonic changes of the model map with SNR increase (Figure 6) denote that if the SNR is sufficiently high, the optimal model is decided by the signal and not by the noise. If noise governs the optimal model selection in the NA6 data, the curves will not be smooth and monotonic. An additional point is the tendency of the curves reaching a steady state with SNR increase. Considering that not only the SNR but also the similarity of the averaged data to the NA6 data increases upon increasing the number of averages, both factors can be the causes of the tendency. However, either of them indicates that the signal is dominant, rather than noise, at optimum model selection. Third, minimal inter-subject variability demonstrates the high reproducibility of the proposed method. Table 2 shows that the SD of each parameter obtained with the optimal model is almost the same as that of the other models although the mean values vary among models. If the optimal model is selected by chance, the SD should be much larger since the ratio of the mixture of the three models varies among the subjects so that the resultant parameter for each subject differs among the subjects.

In the IVIM theory, *f*_*p*_ is a CBV-correlated parameter that could be a surrogate marker for evaluating the pathology of capillaries. However, some previous studies have shown higher GM/WM ratios of *f*_*p*_ than that of CBV by referring to the knowledge of brain perfusion (Wirestam et al., 2001; Fujima et al., 2014; Wu et al., 2015; Shen et al., 2016). Additionally, compared with the Kurtosis model, applying the Gaussian model to brain tissues can yield *f*_*p*_ values approximately twice as high as those of GM (Pavilla et al., 2017). Previously, the correlation of D^*^ to MTT^−1^ has been argued (Wirestam et al., 2001; Fujima et al., 2014; Wu et al., 2015; Shen et al., 2016). With the optimal modeling method (Table 2), the estimated *f*_*p*_ values of GM and WM were 8.3 and 5.4%, respectively, and the GM/WM ratio of *f*_*p*_ was 1.53; the estimated D^*^ values of GM and WM were 6.7 and 5.4 μm^2^/ms, respectively, and the GM/WM ratio of D^*^ was 1.24. These values were reasonable relative to those of previous perfusion studies (Leenders et al., 1990; Shin et al., 2007; Carroll et al., 2008).

In previous studies, the estimated *K* was expected to be lower in GM than in WM. (Jensen et al., 2005; Jensen and Helpern, 2010) With the proposed method, i.e., the optimal model map, the estimated *K* values of GM and WM were closer to each other compared with the single model fittings (Figure 5 and Table 2). The results indicated that inadequate diffusion fitting might cause overestimation or underestimation of *K*. Before applying *K* to assess correlations with the pathological information, the meaning of *K* should be carefully investigated.

Some limitations should be noted in this study. First, the spatial resolution was 3 × 3 × 3 mm^3^, which is close to the cortical thickness in this study. Therefore, only bulk GM voxels could be estimated. Second, to obtain a reliable estimation, high SNR of the IVIM-MRI series with 17 *b*-values was required. The optimal protocols about the b-value distribution and the SNR need to be investigated. The time-consuming issue of data acquisition should be seriously considered in the future. Some novel denoising techniques can gain around 1.4 times SNR for the IVIM data (Huang and Lin, 2019), i.e., half the scan time could be saved by a well-developed denoising process. Some pre- or post-processing techniques, such as Bayesian shrinkage prior and machine learning, may also improve the quantitative uncertainties (Neil and Bretthorst, 1993; Oshio et al., 2014; Clayden et al., 2016; Zhang et al., 2019). Third, to avoid the asymmetric diffusion effect, the data from six MPG directions were analyzed direction-by-direction. However, the perfusion was expected to be symmetric. Validation using the data from a single MPG direction should be further investigated. In addition, due to the lack of the pathological study, the relationship of the parameters estimated by the optimal model mapping and the traditional perfusion techniques were not clear. The results need to be verified with other modalities in the future.

In conclusion, an optimal model mapping method for IVIM-MRI to improve quantification of brain perfusion was proposed. When compared with the conventional methods, the proposed method provided estimated *f*_*p*_ and D^*^ values that were reliable and reproducible. Besides, the optimal model maps might give additional information for clinical diagnosis. In future research, we plan to apply this method to patients and compare the results with those of other methods.

## Data Availability Statement

The original contributions presented in the study are included in the article/Supplementary Material, further inquiries can be directed to the corresponding author.

## Ethics Statement

The studies involving human participants were reviewed and approved by Ethics Committee Graduate School and Faculty of Medicine Kyoto University. The patients/participants provided their written informed consent to participate in this study.

## Author Contributions

Y-PL and S-iU: conception or design of the work, data collection, data analysis and interpretation, drafting the article. TI and HF: critical revision of the article, final approval of the version to be published. All authors contributed to the article and approved the submitted version.

## Conflict of Interest

The authors declare that the research was conducted in the absence of any commercial or financial relationships that could be construed as a potential conflict of interest.

## Supplementary Material

Supplementary Figure 1 The optimal model maps for six subjects with different NAs, where the colors indicated the optimal model (blue for Gaussian model, green for Kurtosis model, and yellow for Gamma model).
